# Accuracy and Robustness of the Interpolation Cover Method for Structural Analysis: Effects of Neighboring-Node Selection and Polynomial Enrichment

**DOI:** 10.3390/ma19214669

**Published:** 2026-11-02

**Authors:** Waseem Arif, Sajjad Miran, Tayybah Kiren, Naeem Ullah

**Affiliations:** 1 Department of Mechanical Engineering, University of Gujrat, Gujrat 50700, Pakistan; sajjad.miran@uog.edu.pk; 2 Department of Computer Science, Rcet Campus, University of Engineering and Technology, Lahore 54890, Pakistan; tayybah@uet.edu.pk; 3 Department of Mechanical Engineering Technology, Punjab Tianjin University of Technology, Lahore 54770, Pakistan

**Keywords:** interpolation covers method, enrichment, solution quality, mesh dependency, polynomial degree of freedom

## Abstract

This paper aims to analyze the performances of the interpolation covers method in the enrichment of a standard four-node quadrilateral finite element (FE) solution. The interpolation covers (IC) method has shown to be efficient in the local enrichment of the solution by increasing the polynomial degree of approximation using neighboring elements within a predefined set of covers. The enriched FE approximation is capable of capturing high stress gradients accurately, even using coarse mesh, thereby reducing the computational cost. As reported in existing studies, researchers have used neighboring nodes and polynomial enrichment to construct interpolation covers, but the combined effect of the selection of the number of neighboring nodes and their position in the patches and polynomial enrichment has not explored been yet. This study therefore performs a parametric investigation of these factors for enriched four-node, quadrilateral-shell finite elements. Two numerical applications are treated to demonstrate the effectiveness of the method. It is found that the selection of the closest neighboring nodes results in high solution accuracy.

## 1. Introduction

Achieving predictive computational finite element models, especially for stress approximation, requires either the use of higher order finite elements or the use of mesh refinement by subdividing the element sizes. Unfortunately, both techniques lead to high computational cost and, hence, become unfeasible for complex geometries [1].

A noticeable body of research has been published in the past few years to improve the FE solution. The smoothed FE technique was proposed by Chen et al. [2] to solve various engineering problems in which the constant strain fields were constructed in newly defined smoothing domains [3]. The latter could be defined as node-based, cell-based, edge-based, or face-based smoothed FE. Each technique had different characteristics and performance depending on the smoothing domain [4,5,6,7]. Dreàu et al. [8] used the XFEM with higher order FE enrichment functions to track the material interface so that curved geometries could be represented implicitly. Agathos et al. [9] introduced a simple procedure in the XFEM for the local near-orthogonalization of enrichment functions, which significantly improved the conditioning of the resulting system matrices with minor modifications to the initial method. Leconte et al. [10,11] developed a “super element” based on a hybrid-Trefftz variational principle to model riveted joints. They showed good stress fields in comparison with the standard FE for elastic materials. Other research investigations [12,13,14,15] have been reported based on the development of new elements to identify singular stresses. In particular, Icardi et al. [15] proposed a mixed singular wedge element with a variable singularity power to model multilayered composites. They showed the ability to adapt the variable singularity power according to the problem and represent non-singular behavior as a particular case for analysis of damaged composites. The effectiveness of the method was illustrated through sample cases with singular stress fields due to dissimilar properties of materials, geometry or cracks, for which analytical solutions are available. Payen et al. [16] presented a stress improvement procedure based on the mixed variational formulation to solve static, dynamic and nonlinear problems. The authors of [17,18] discussed the use of nodal point forces to improve element stresses at low computational cost. The effectiveness of their method was illustrated through the solution of several benchmarks. Kim et al. [19] presented continuum mechanics-based beam elements for linear and nonlinear analyses of multilayered composite beams with inter-layer slips. The proposed beam FE was successfully applied in predicting geometric and material nonlinear behaviors of multilayered beams, including nonlinear load-slip relations at inter layers.

Shi [20,21] proposed a Manifold Numerical Method (MNM) where different displacement functions are utilized on material domains. Both FE for continuous and Discontinuous Deformation Analysis (DDA) for block systems are special cases of the MNM [22]. Zhang et al. and Ma et al. [23,24] used the MNM to solve crack propagation problems within the material domain. The main limitation of the MNM is that this technique suffers from a rank-deficient global matrix.

By using the idea of the MNM, Bathe et al. [25] introduced the Interpolation Covers (IC) technique for FE enrichment. It is based on the use of IC functions, along with standard finite element shape functions, to improve the solution accuracy of low-order finite elements). To illustrate the effectiveness of the method, three-node triangular and four-node tetrahedral elements were selected to mesh simple geometries, and results were discussed [25]. Kim et al. [26] proposed new four-node quadrilateral FE, eight-node hexahedral, six-node prismatic and five-node pyramidal 3D solid FE [27] enriched by IC functions. Rank deficiencies of the stiffness matrix and linear dependence problem were resolved by adopting piecewise linear shape functions as the partition of unity functions. The convergence behavior and effectiveness of the new enriched elements and adaptive use of cover functions were demonstrated by solving a number of numerical problems. In another study, Lee et al. [28] proposed an automatic procedure to improve the solution accuracy using enriched 2D solid FE. An error for each node in the given meshes was estimated; then, proper degrees of cover functions were applied to the selected nodes. This study was extended by Oh et al. [29] to present an automatic procedure that enhances the solution accuracy of edge-based smoothed 2D solid FE. The IC method was also used to improve the FE displacement and stress of MITC3 shell elements by Jeon et al. [1]. Kim and Bathe [30] then proposed the automatic use of IC to improve the finite element solution. This enrichment scheme was employed to improve the membrane displacement of an MITC+3 shell element [31]. Lee et al. [32] suggested strain-smoothed MITC3+ shell FE. The membrane behavior of the MITC3+ shell FE was significantly improved by using the strain-smoothed element (SSE) method without additional degrees of freedom. The method decreased the inter-element stress jumps and could be employed for distorted elements. The interpolation covers method is quite flexible in that a variety of cover functions can be used, depending upon the solution accuracy needed to improve in different patches without mesh refinement. However, rank deficiency problems can also be encountered as in meshless methods [25,30,31]. Chai, Li and Liu [33] developed an enriched FE method in which conventional linear triangular shape functions are supplemented with Lagrangian and harmonic trigonometric interpolation-cover functions. Their analysis showed that the approach can substantially reduce spatial dispersion while retaining the simplicity of low-order triangular elements. An important contribution was also the treatment of the linear-dependence problem associated with enrichment. Qu et al. [34] proposed an appropriate interpolation-cover function for transient wave propagation and investigated the influence of enrichment order and numerical parameters. The research demonstrates a broader movement toward combining conventional finite elements with spectral-like enrichment functions. Bathe and Zhang [35] proposed the overlapping of finite elements as a way of reducing the difficulty of generating high-quality meshes for complicated geometries. Instead of requiring conventional elements to fit together perfectly, overlapping regions can be used to cover the computational domain. Zhang, Kim and Bathe [36] subsequently investigated the computational efficiency of this approach and emphasized the use of local polynomial interpolation to obtain accurate solutions with reduced sensitivity to mesh distortion. Huang and Bathe [37] provided a more theoretical investigation of convergence of overlapping elements and overlapping meshes, showing that these approaches can provide accurate solutions while reducing the dependence on highly optimized conventional meshes. Lee, Yun and Kim [38] addressed an important limitation of earlier interpolation-cover formulations: although enrichment improves accuracy, applying high-order enrichment throughout the entire model can produce a large number of degrees of freedom and consequently high computational cost.

Previous studies show that neighboring nodes and polynomial enrichment in interpolation covers enhance the solution accuracy without mesh refinement. But the combined effect of the number and spatial selection of neighboring nodes with the polynomial degree has received comparatively limited systematic investigation, particularly for the enrichment of standard four-node quadrilateral-shell finite elements. The present study addresses this issue through a systematic parametric investigation of the combined influence of the selection of the number and position of neighboring nodes with a polynomial degree of enrichment using interpolation covers on the accuracy and robustness of the ICM when enriching standard four-node quadrilateral-shell finite elements. Basic formulation of the IC method is briefly discussed. The results obtained using the method are illustrated by two benchmark problems. They demonstrate the effectiveness of the mentioned enrichment approach when comparing the results with standard FEM. This enrichment approach is also used to determine the influence of selection of the neighboring nodes and selection of the polynomial degree of enrichment on the solution accuracy. Finally, concluding remarks are discussed.

## 2. Enrichment Using the Interpolation Covers Method

**Figure 1:**
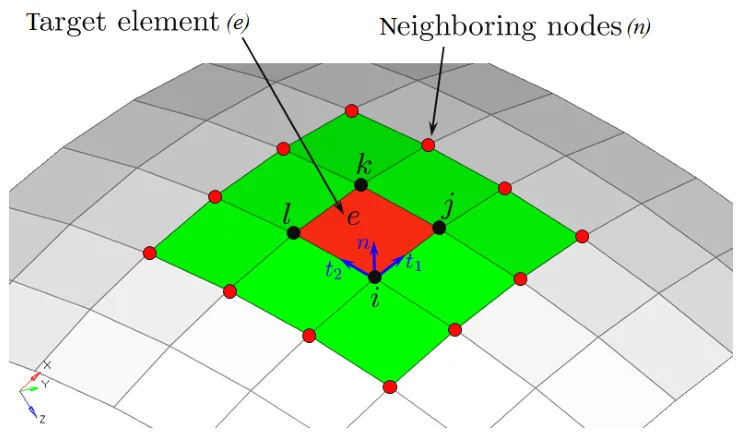
Description of sub-domains for enriched cover interpolations [39].

FEM is an effective numerical approach to solve physical problems. However, the selection of the proper mesh type, as well as the number and type of elements, is challenging in obtaining the desired solution accuracy. Sometimes, very fine FE mesh schemes are adopted to ensure reliable solution accuracy, leading to undesired high computational cost. To avoid this, we used the interpolation covers method to improve the solution accuracy of the standard low-order FE discretization without any increase in the number of elements. In the interpolation covers method, higher order enrichment can be achieved without introducing the additional degrees of freedom (DOFs) through the use of surrounding nodes belonging to the target shell element (“*e*”), as shown in Figure 1.

Let $Q^{n}={\left\{x_{i}\right\}}_{i=1}^{n}$ be a set of position vectors of *n* enlisted surrounding nodes presented in the local framework ($t_{1},t_{2},n$) of the target element (*e*) given by

(1) $$x_{i}=Q^{T}\;X_{i}$$

where $X_{i}$ is the position vector of node *i* surrounding the target element (*e*) and $Q=\left[t_{1}\;\vdots \;t_{2}\;\vdots \;n\right]$ is the orthonormal basis of the target element (*e*). To enrich the standard FE interpolation for the solution variable (*x*), we use interpolation cover functions, i.e.,

(2) $$P_{i}^{p}\left[u\right]=u_{i}+{\left\{\xi ,\;\eta ,\;\xi^{2},\;\xi \eta ,\;\eta^{2},\;\cdots ,\;\eta^{p}\right\}}^{T}{\hat{u}}_{i}$$

where $u_{i}$ represents the standard quadrilateral nodal DOF, ${\hat{u}}_{i}=\{{\hat{u}}_{i}^{\xi },\;{\hat{u}}_{i}^{\eta },\;{\hat{u}}_{i}^{\xi_{i}^{2}},\;{\hat{u}}_{i}^{\xi_{i}\eta_{i}},\;{\hat{u}}_{i}^{\eta_{i}^{2}},\;\cdots ,$$\;{\hat{u}}_{i}^{\eta_{i}^{p}}{\}}^{T}$ lists additional degrees of freedom per cover region and *p* shows the order of the complete polynomial used.

The enriched cover approximation of the field variable (*u*) is represented by

(3) $$u=\sum_{e=1}^{nelt}\sum_{i\in i_{c}\left(e\right)}N_{i}\;P_{i}^{p}\left[u\right]=\sum_{e=1}^{nelt}\left(\sum_{i\in i_{c}\left(e\right)}N_{i}\;u_{i}+\sum_{i\in i_{c}\left(e\right)}{\tilde{N}}_{i}\;{\hat{u}}_{i}\right)$$

with $i_{c}\left(e\right)$ representing the set of cover indices and ${\tilde{N}}_{i}$ denoting the enriched higher degree shape function, i.e.,

(4) $${\tilde{N}}_{i}=N_{i}\;{\left\{\xi ,\;\eta ,\;\xi^{2},\;\xi \eta ,\;\eta^{2},\;\cdots ,\;\eta^{p}\right\}}^{T}$$

Considering Equation (4), the enriched cover approximation consists of the standard FE interpolation plus additional higher order terms. To obtain a well-conditioned stiffness matrix, we use the local parametric coordinate systems (ξ,η) of the target element instead of the Cartesian coordinates (x,y).

If $P_{i}^{p}\left[u\right]=u_{i},$ then the scheme reduces to the standard linear interpolation. Indeed, we can regard the enriched interpolation as the standard finite element interpolation plus additional higher order terms.

An arbitrary degree of polynomial bases can be adopted in the cover interpolations. However, since high-order covers yield more unknowns, we use up to quadratic covers in this work, i.e., we shall use p≤2.

### 2.1. Interpolation Covers Method Applied to the Quadrilateral Shell Element

The standard FE approximation of the solution of the four-node shell element is

(5) $$\delta u_{q}=\delta u+z\;\delta \beta =\sum_{i=1,4}N_{i}\;\delta u_{i}+z\;\sum_{i=1,4}N_{i}\;\left(-t_{2i}\;\delta \theta_{1i}+t_{1i}\;\delta \theta_{2i}\right)$$

where $N_{i}\left(\xi ,\eta \right)$ are the planar shape functions of the Q4 shell element, i.e.,

(6) $$N_{i}\left(\xi ,\eta \right)=\frac{1}{4}\left(1+\xi_{i}\;\xi \right)\left(1+\eta_{i}\;\eta \right);\;i=1,4$$

where $\delta u={\left\{\delta U,\delta V,\delta W\right\}}^{T}$ is the virtual translational vector and $\delta \beta ={\left\{\delta \theta_{1},\delta \theta_{2}\right\}}^{T}$ is the virtual rotational vector, which can be written in Cartesian components.

Following Equation (3), the virtual components of displacements (δU,δV,δW) and rotations ($\delta \theta_{1},\delta \theta_{2}$) can be enriched using the ICM over the set of covers of target element *e* as follows:

(7) $$\delta U^{\left(e\right)}=\sum_{i\in i_{c}\left(e\right)}\left(N_{i}\;\delta U_{i}+{\tilde{N}}_{i}\;\delta {\hat{U}}_{i}\right);\;\mathrm{same}\;\mathrm{for}:\;\delta V^{\left(e\right)},\delta W^{\left(e\right)},\delta \theta_{1}^{\left(e\right)},\delta \theta_{2}^{\left(e\right)}$$

where $\delta U_{i}$ represents the usual four nodal values at the fournodes of the quadrilateral shell element and the $\delta {\hat{U}}_{i}$ is the list of additional unknown DOFs belonging to the *n* neighboring nodes.

For linear covers, we need to put p=1 in Equation (4), this will enrich the shell element from standard bilinear approximation of Equation (6) to a quadratic approximation; then, we have

(8) $${\tilde{N}}_{i}=N_{i}\;{\left\{\xi ,\;\eta \right\}}^{T},$$

and for quadratic covers, according to Equation (4), using p=2 we also have

(9) $${\tilde{N}}_{i}=N_{i}\;{\left\{\xi ,\;\eta ,\;\xi^{2},\;\xi \eta ,\;\eta^{2}\right\}}^{T}$$

The finite element approximation of virtual displacements for the full quadrilateral four-node Q4 shell element leads to the matrix form, using nodal DOFs at four nodes and some of the surrounding nodes.

(10) $$\delta u_{q}=N\;\delta u_{n}+\tilde{N}\;\delta {\hat{u}}_{n}$$

with

(11) $$\begin{array}{c} \delta u_{n}={\left\{\ldots ,\delta U_{i},\delta V_{i},\delta W_{i},\delta \theta_{1i},\delta \theta_{2i},\ldots \right\}}^{T};\\ N_{3\times 20}=\left[\begin{array}{ccccccc} \cdots & \begin{array}{c} ¦\\ ¦\\ ¦\\ ¦ \end{array} & \begin{array}{ccc} N_{i} & 0 & 0\\ 0 & N_{i} & 0\\ 0 & 0 & N_{i} \end{array} & \begin{array}{c} ¦\\ ¦\\ ¦\\ ¦ \end{array} & \begin{array}{c} zN_{i}{V_{X}}^{T}\\ zN_{i}{V_{Y}}^{T}\\ zN_{i}{V_{Z}}^{T} \end{array} & \begin{array}{c} ¦\\ ¦\\ ¦\\ ¦ \end{array} & \cdots \end{array}\right];\;i=1,4\\ V_{X}={\left\{-t_{2Xi},\;t_{1Xi}\right\}}^{T};\;V_{Y}={\left\{-t_{2Yi},\;t_{1Yi}\right\}}^{T};\;V_{Z}={\left\{-t_{2Zi},\;t_{1Zi}\right\}}^{T} \end{array}$$

If we use linear covers of Equation (8), then we need two extra surrounding nodes numbered here as 5 and 6; therefore, the enrichment matrices are expressed as follows:

(12) $$\begin{array}{c} \delta {\hat{u}}_{n}={\left\{\cdots ,\delta U_{j},\delta V_{j},\delta W_{j},\delta \theta_{1j},\delta \theta_{2j},\cdots \right\}}^{T};\\ {\tilde{N}}_{3\times 10}=\left[\begin{array}{ccccccc} \cdots & \begin{array}{c} ¦\\ ¦\\ ¦\\ ¦ \end{array} & \begin{array}{ccc} {\tilde{N}}_{j} & 0 & 0\\ 0 & {\tilde{N}}_{j} & 0\\ 0 & 0 & {\tilde{N}}_{j} \end{array} & \begin{array}{c} ¦\\ ¦\\ ¦\\ ¦ \end{array} & \begin{array}{c} z{\tilde{N}}_{j}{V_{X}}^{T}\\ z{\tilde{N}}_{j}{V_{Y}}^{T}\\ z{\tilde{N}}_{j}{V_{Z}}^{T} \end{array} & \begin{array}{c} ¦\\ ¦\\ ¦\\ ¦ \end{array} & \cdots \end{array}\right];\;j=5,6\\ V_{X}={\left\{-t_{2Xj},\;t_{1Xj}\right\}}^{T};\;V_{Y}={\left\{-t_{2Yj},\;t_{1Yj}\right\}}^{T};\;V_{Z}={\left\{-t_{2Zj},\;t_{1Zj}\right\}}^{T} \end{array}$$

Using properly arranged unknowns in vectors, the enriched virtual displacement vector takes the following expression:

(13) $$\delta u_{q}={\left[N\;\vdots \;\tilde{N}\right]}_{\left(3\times 30\right)}\;{\left\{\begin{array}{c} \delta u_{n}\\ \delta {\hat{u}}_{n} \end{array}\right\}}_{\left(30\times 1\right)}=\bar{N}\;\delta {\bar{u}}_{n}$$

$\bar{N}$ are the new augmented enriching shape functions, which are obtained for the enriched target element (*e*), which now uses 30 degrees of freedom (in the case of linear covers), resulting from adding 10 extra DOFs of two surrounding nodes.

Once $\bar{N}$ are obtained, then the standard procedure of generating the FE matrices for the virtual strains remains the same as shown previously. The only difference is the replacement of N with the new augmented shape functions ($\bar{N}$).

The internal force vector remains exactly the same by substituting B matrices with $\bar{B}$ as follows:

(14) $$\begin{array}{cc} \;f_{int}^{e}= & \int_{\xi =-1}^{\xi =+1}\int_{\eta =-1}^{\eta =+1}\int_{\zeta =-1}^{\zeta =+1}\left({\bar{B}}_{o}^{T}+\frac{h}{2}\zeta \;{\bar{B}}_{1}^{T}+\frac{h^{2}}{4}\zeta^{2}\;{\bar{B}}_{2}^{T}\right)\sigma_{s}\;J_{o}\;\frac{h}{2}\;d\zeta \;d\eta \;d\xi \;+\\ \; & \int_{\xi =-1}^{\xi =+1}\int_{\eta =-1}^{\eta =+1}\int_{\zeta =-1}^{\zeta =+1}\left({\bar{B}}_{so}^{T}+\frac{h}{2}\zeta \;{\bar{B}}_{s1}^{T}\right)\tau \;J_{o}\;\frac{h}{2}\;d\zeta \;d\eta \;d\xi \end{array}$$

Contrarily to the standard formulation, now the internal force vector given in Equation (14) is integrated in the mid-surface (ξ,η) using $n_{g}\times n_{g}$ Gaussian quadrature points. Where $n_{g}$ is the number of integration points, its value has to be fixed by the user. For linear covers, a value of $n_{g}=3$ has been found to be a good value for stable results. When using higher degree covers, then the value of $n_{g}$ has to be high, resulting in an increase in CPU computing time.

Integration through the thickness direction (ζ) is still achieved using five or more Lobatto quadrature points, depending on the degree of plastic strains and the desired accuracy.

It has to be noted that the mass matrix of the enriched element remains the same as the standard form. Therefore, no enrichment was performed for the evaluation of the mass matrix. The same procedure is also applied to generate the lumped mass matrix.

### 2.2. Assembly Procedure for the Enriched Shell Element

In the following, the general special procedure needed for the assembly of the internal force vector of enriched elements is presented. Indeed, as a result of the introduction of extra nodes surrounding the target enriched element, the resulting internal force vector depends on the DOFs of its four standard nodes, as well as on the surrounding nodes used for the enrichment.

**Figure 2:**
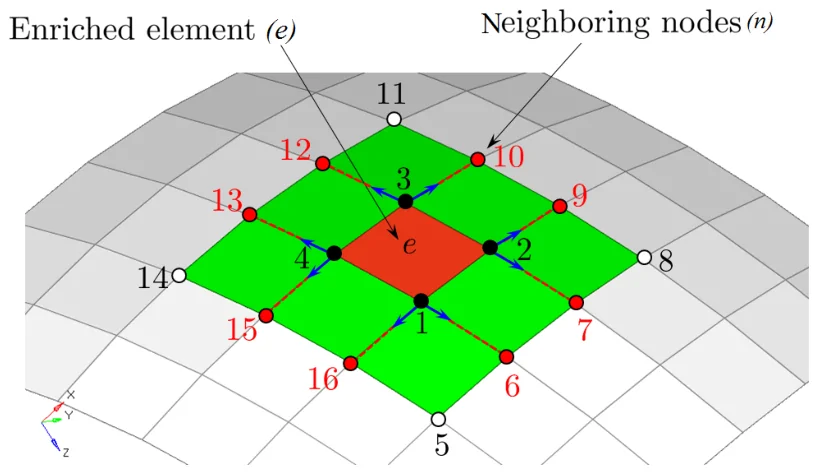
Assembly procedure for the enriched shell element [39].

Let us consider a target element (*e*) that has to be enriched using surrounding extra nodes, as indicated in Figure 2. Suppose that the target element has nodes 1, 2, 3 and 4, assuming that surrounding nodes $U_{6},U_{7},U_{9},U_{10},U_{12},U_{13},U_{15}$ and $U_{16}$ are used for enrichment. In this case and following Equations (7) and (8), the new enriched FE approximation reads

(15) $$\delta U^{\left(e\right)}=\sum_{i\in i_{c}\left(e\right)}\left(N_{i}\;\delta U_{i}+{\tilde{N}}_{i}\;\delta {\hat{U}}_{i}\right);\;\mathrm{same}\;\mathrm{for}:\;\delta V^{\left(e\right)},\delta W^{\left(e\right)},\delta \theta_{1}^{\left(e\right)},\delta \theta_{2}^{\left(e\right)}$$

where $\delta U_{i}$ represents the usual four nodal values at the fournodes of the quadrilateral shell element and $\delta {\hat{U}}_{i}$ is the list of additional unknown DOFs belonging to the *n* neighboring nodes.

Using linear covers (${\tilde{N}}_{i}=N_{i}\;{\left\{\xi ,\;\eta \right\}}^{T}$) to enrich the shell element from standard bilinear approximation of Equation (6) to a quadratic approximation, Equation (15) can be written explicitly for the particular example of Figure 2 as follows:

(16) $$\begin{array}{cc} \;\delta U^{\left(e\right)}= & N_{1}\;\delta U_{1}+N_{2}\;\delta U_{2}+N_{3}\;\delta U_{3}+N_{4}\;\delta U_{4}+\\ \; & N_{1}\;\xi \;\delta U_{6}+N_{1}\;\eta \;\delta U_{7}+N_{2}\;\xi \;\delta U_{9}+N_{2}\;\eta \;\delta U_{10}+\\ \; & N_{3}\;\xi \;\delta U_{12}+N_{3}\;\eta \;\delta U_{13}+N_{4}\;\xi \;\delta U_{15}+N_{4}\;\eta \;\delta U_{16} \end{array}$$

In Equation (16), $U_{1},\ldots ,U_{4}$ are standard DOFs of the quadrilateral shell element, and $U_{6},U_{7},U_{9},U_{10},U_{12}$, $U_{13},U_{15}$ and $U_{16}$ are extra DOFs of surrounding nodes, as depicted in Figure 2.

The FE approximation used for the first displacement component ($\delta U^{\left(e\right)}$) in Equation (16) can be repeated for the remaining four kinematic field components—namely, $\delta V^{\left(e\right)},\delta W^{\left(e\right)},\delta \theta_{1}^{\left(e\right)}$ and $\delta \theta_{2}^{\left(e\right)}$. As a result, for the case of enriched element (e) given in Figure 2, one can have 8×5=40 extra DOFs. Knowing that the standard quadrilateral shell element has 5×4=20 DOFs, as a result, the enriched element will have a total of 40+20=60 DOFs in the local framework; after transformation to the global framework by adding the $\theta_{z}$ DOF, it increases to a total of 48+24=72 DOFs.

Let us now consider the expression of the internal virtual work of a standard quadrilateral shell element, which we can write as follows:

(17) $$\begin{array}{cc} \;W_{int}^{e}= & \underset{1\times 24}{\underset{︸}{\delta u_{n}^{T}}}\;\underset{24\times 1}{\underset{︸}{F_{int}^{e}}} \end{array}$$

where $u_{n}$ is the vector of global DOFs of size 24×1, given by $u_{n}={\left\{\ldots ,U_{i},V_{i},W_{i},\theta_{Xi},\theta_{Yi},\theta_{Zi},\ldots \right\}}^{T}$ for i=1,4.

The internal virtual work of the enriched element of Figure 2 will be written as

(18) $$\begin{array}{cc} \;W_{int}^{e}= & \underset{1\times 24}{\underset{︸}{\delta u_{n}^{T}}}\;\underset{24\times 1}{\underset{︸}{F_{int}^{e}}}+\underset{1\times 48}{\underset{︸}{\delta {\hat{u}}_{n}^{T}}}\;\underset{48\times 1}{\underset{︸}{{\hat{F}}_{int}^{e}}} \end{array}$$

In a symbolic writing by showing matrix positions of each node, the standard internal force vector maybe written as follows:

(19) $$\begin{array}{cc} \;\underset{1\times 24}{\underset{︸}{{\left(F_{int}^{e}\right)}^{T}}}= & \left\{\underset{1\times 6}{\underset{︸}{{\left(F_{1}\right)}^{T}}},\underset{1\times 6}{\underset{︸}{{\left(F_{2}\right)}^{T}}},\underset{1\times 6}{\underset{︸}{{\left(F_{3}\right)}^{T}}},\underset{1\times 6}{\underset{︸}{{\left(F_{4}\right)}^{T}}}\right\} \end{array}$$

where $F_{1},\ldots ,F_{4}$ are nodal internal force vectors. In the same manner, the internal force vector of surrounding nodes can be written as follows:

(20) $$\begin{array}{cc} \;\underset{1\times 48}{\underset{︸}{{\left({\hat{F}}_{int}^{e}\right)}^{T}}}= & \left\{\underset{1\times 6}{\underset{︸}{{\left(F_{6}\right)}^{T}}},\underset{1\times 6}{\underset{︸}{{\left(F_{7}\right)}^{T}}},\underset{1\times 6}{\underset{︸}{{\left(F_{9}\right)}^{T}}},\underset{1\times 6}{\underset{︸}{{\left(F_{10}\right)}^{T}}},\underset{1\times 6}{\underset{︸}{{\left(F_{12}\right)}^{T}}},\underset{1\times 6}{\underset{︸}{{\left(F_{13}\right)}^{T}}},\underset{1\times 6}{\underset{︸}{{\left(F_{15}\right)}^{T}}},\underset{1\times 6}{\underset{︸}{{\left(F_{16}\right)}^{T}}}\right\} \end{array}$$

For the assembly procedure, let us consider the patch of elements and nodes from 1,…,16 as shown in Figure 2. The corresponding global internal force vector of size 16×6=96, which results from the assembly procedure, is always stored in chronological order of nodes 1, 2, 3, …, 16.

Therefore, during the assembly process of enriched element (e), one has to localize the right number of equations corresponding to each extra DOF in the global table, which can be represented as follows:

(21) $$\begin{array}{cc} \;\underset{1\times 96}{\underset{︸}{{\left(F_{int}\right)}^{T}}}= & \{\underset{1\times 6}{\underset{︸}{{\left(F_{1}\right)}^{T}}},\underset{1\times 6}{\underset{︸}{{\left(F_{2}\right)}^{T}}},\underset{1\times 6}{\underset{︸}{{\left(F_{3}\right)}^{T}}},\underset{1\times 6}{\underset{︸}{{\left(F_{4}\right)}^{T}}},\underset{1\times 6}{\underset{︸}{0}},\underset{1\times 6}{\underset{︸}{{\left(F_{6}\right)}^{T}}},\underset{1\times 6}{\underset{︸}{{\left(F_{7}\right)}^{T}}},\underset{1\times 6}{\underset{︸}{0}},\\ \; & \underset{1\times 6}{\underset{︸}{{\left(F_{9}\right)}^{T}}},\underset{1\times 6}{\underset{︸}{{\left(F_{10}\right)}^{T}}},\underset{1\times 6}{\underset{︸}{0}},\underset{1\times 6}{\underset{︸}{{\left(F_{12}\right)}^{T}}},\underset{1\times 6}{\underset{︸}{{\left(F_{13}\right)}^{T}}},\underset{1\times 6}{\underset{︸}{0}},\underset{1\times 6}{\underset{︸}{{\left(F_{15}\right)}^{T}}},\underset{1\times 6}{\underset{︸}{{\left(F_{16}\right)}^{T}}}\} \end{array}$$

The localization number of the starting internal force vector of surrounding node number 6 is, in this case, 31=5×6+1; for example, the localization number of the starting internal force vector of surrounding node number 15 is, in this case, 85=14×6+1.

The above assembly procedure for enriched elements was developed for the general case and was implemented in an in-house code.

In the present paper, the interpolation covers method is used to solve exclusively static structural applications so that the mass matrix does not affect the solution accuracy in terms of stress, strain and displacement because the mass matrix does not contribute to the equilibrium equation. For simplicity, the conventional finite-element mass matrix is consequently retained. For dynamic or eigenvalue analysis, however, this assumption requires further consideration. Since the interpolation cover enriches the displacement approximation, a consistent dynamic formulation should derive the mass matrix from the complete enriched displacement field. In general, the corresponding mass matrix contains contributions from both the conventional and enriched approximation functions, including their coupling terms. Retaining the standard mass matrix in such an application may therefore introduce discrepancies in the computed natural frequencies, mode shapes, or transient responses. The standard mass matrix adopted in the present work is intended only for the static formulation considered here and should not be regarded as a validated mass formulation for dynamic ICM analysis. A consistent enriched mass matrix and its effects on modal and transient responses require further investigation.

### 2.3. Assembly of Multiple Enriched Elements with Overlapping Covers

In practice, interpolation covers associated with different enriched elements can overlap, and several enriched elements can share the same neighbor nodes. The situation is illustrated in Figure 1 for two enriched elements with interpolation covers that partially overlap. In the current implementation, the problem of overlapping interpolation covers is addressed by utilizing a unique degree-of-freedom numbering procedure for all enriched elements. In particular, enriched degrees of freedom associated with a particular neighbor node and enrichment order are uniquely identified by their indices; consequently, enriched elements that share the same neighbor nodes have some degrees of freedom in common. For each enriched element, the local stiffness matrix is assembled using both standard and interpolation-cover degrees of freedom and a local-to-global mapping array. The element matrix is then assembled into the global stiffness matrix according to the local-to-global mapping. As a result, if multiple enriched elements share the same neighbor node, their contributions are added to the same degree-of-freedom locations in the global stiffness matrix. In this way, overlapping interpolation covers can be used to enrich finite-element approximations while ensuring that neighbor elements are coupled correctly through shared degrees of freedom. The assembly procedure follows the same procedure as in standard finite element analysis, with the exception that the local degree-of-freedom arrays also include enrichment degrees of freedom. Using a unique numbering for enrichment degrees of freedom and local-to-global mapping ensures that multiple enriched elements that share the same neighbor node and enrichment parameters do not have independent, equal degrees of freedom but use the same global degree-of-freedom indices. Additionally, the same local-to-global mapping is used for all enriched elements such that overlapping interpolation covers generate identical contributions to the global stiffness matrix.

## 3. Results and Discussions

Some numerical academic applications are presented here to show the effectiveness of the ICM. Results will be compared to those obtained using reference analytical solutions and to those obtained using refined mesh solutions of LS DYNA commercial code.

### 3.1. Plate with a Hole

**Figure 3:**
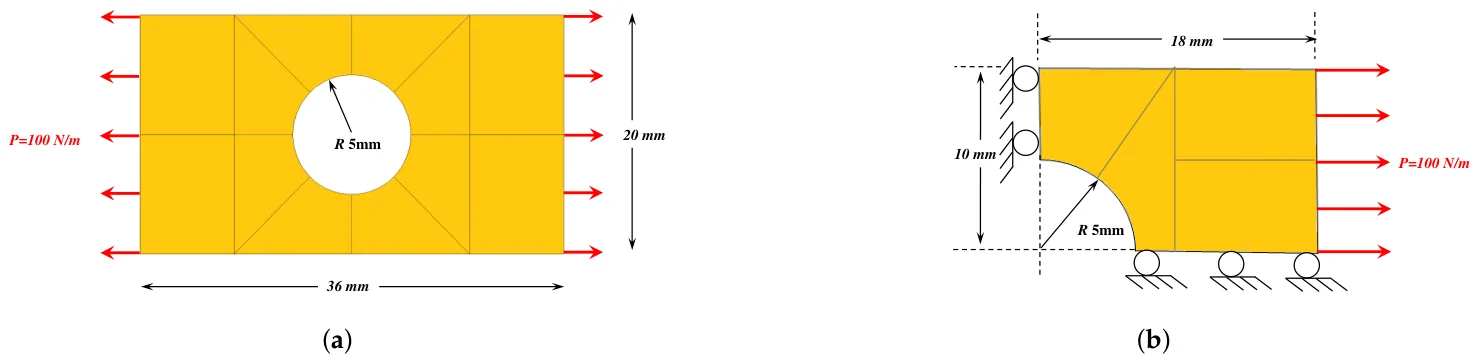
Plate with a central hole under tensile loading [39]. (**a**) Complete geometry with boundary conditions. (**b**) Quarter geometry with symmetry boundary conditions.

The first numerical application concerns a plate with a central hole under tensile loading, as given in Figure 3.

**Figure 4:**
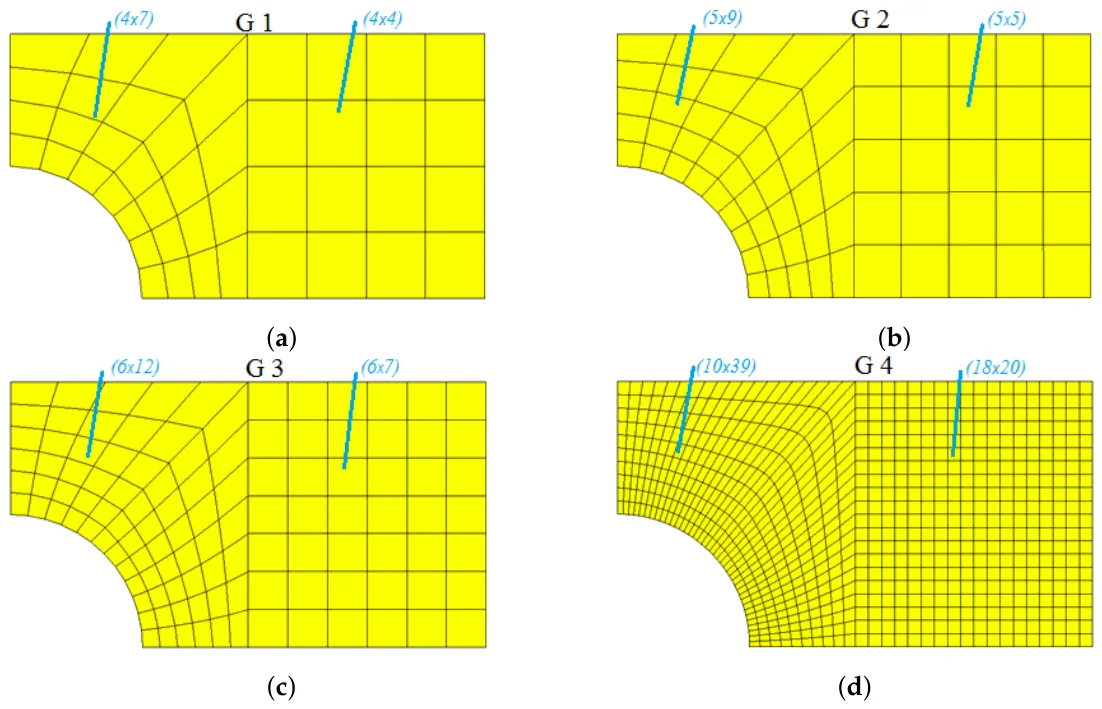
Plate with a hole featuring four different mesh schemes [39]. (**a**) Total number of elements is 44. (**b**) Total number of elements is 70. (**c**) Total number of elements is 114. (**d**) Total number of elements is 750.

A plane stress condition is used with material data corresponding to $E=72\times 10^{9}$ Pa, ν=0, a distributed load of p=100 N/m and thickness taken as unity. In the present numerical examples, a Poisson ratio of ν=0 is adopted as a controlled benchmark assumption. This choice is made to eliminate lateral constitutive coupling and thereby isolate the influence of the interpolation-cover enrichment on the convergence and stress-recovery characteristics. The selected value should not be interpreted as representative of a particular engineering material; rather, it provides a simplified reference configuration for assessing the numerical performance of the proposed formulation. Since volumetric locking is primarily associated with nearly incompressible materials as ν approaches 0.5, the present examples are not intended to investigate locking in the nearly incompressible regime. The interpolation-cover formulation, itself, is not restricted to ν=0 and can be employed with nonzero Poisson ratios for applications involving specific engineering materials. Only a quarter of the plate is modeled due to symmetry. Seven different mesh schemes are opted for to obtain a converged solution, and four of them are shown in Figure 4. There are 74,000, 115,625 and 462,500 elements used to model G5, G6 and G7, respectively. Before evaluating the relative errors of the ICM-enriched elements, a mesh-convergence study was performed using the G6 and G7 meshes in LS-DYNA. The selected response quantities were compared between successive mesh refinements to assess the sensitivity of the benchmark solution to mesh density. The results show that the variation from G6 to G7 is considerably smaller—in the case of a plate with a hole, 0.115%, and in clamped plate bending, 1.55%. That indicates the solution is approaching mesh convergence. On this basis, the G7 solution is adopted as the reference solution for the subsequent error evaluation. It should be noted that G7 is considered a numerically converged benchmark within the mesh sequence examined here rather than an exact analytical solution. Consequently, the relative errors presented subsequently represent deviations from the converged G7 LS-DYNA results.

**Figure 5:**
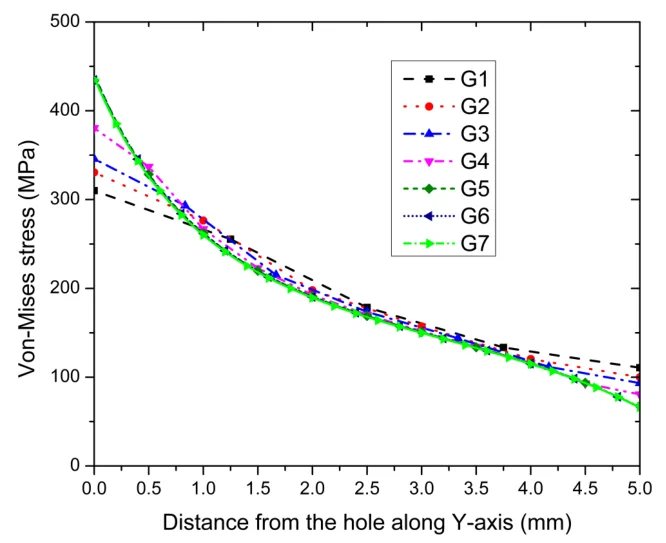
von Mises stress distribution along the Y-axis [39].

The von Mises stress distribution along the Y-axis is plotted in Figure 5 for the different meshes of LS DYNA. We can observe that the converged solution of LS DYNA is obtained for G7; therefore, it will be taken as reference solution for comparison.

**Figure 6:**
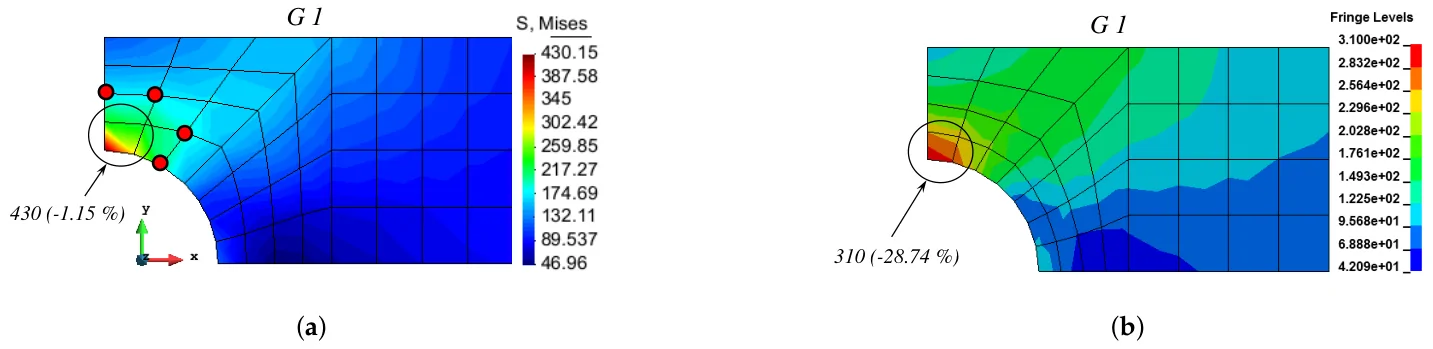
von Mises stress distribution along the Y-axis for G1 [39]. (**a**) ICM solution. (**b**) LS DYNA solution.

**Coarse Mesh Performance (G1):** For the initial coarse mesh discretization (G1) shown in Figure 6, four nearest neighboring nodes were selected to enrich the critical element domain adjacent to the hole boundary (highlighted by the black circle in Figure 6a). While the standard LS-DYNA Q4 element formulation severely underestimated the stress concentration, yielding an error of −28.74%, the ICM formulation achieved remarkable accuracy, with an error of only −1.15% on the exact same mesh (Figure 6b).

**Figure 7:**
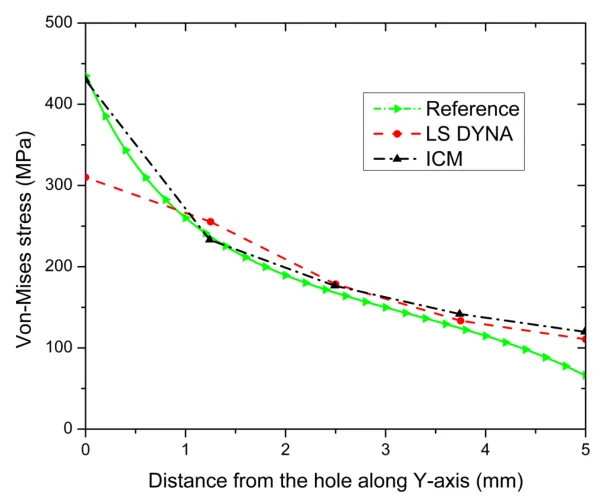
von Mises stress distribution along the Y-axis for G1 [39].

The radial von Mises stress field trajectory plotted along the *Y*-axis from the hole edge further underscores the superior gradient resolution of the ICM relative to the reference curve (Figure 7).

**Figure 8:**
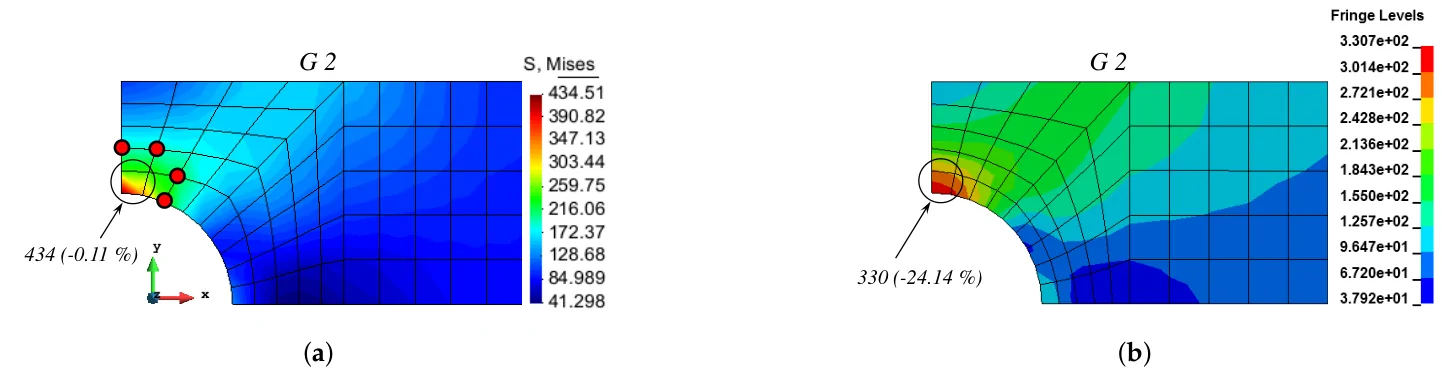
von Mises stress distribution along the Y-axis for G2 [39]. (**a**) ICM solution. (**b**) LS DYNA solution.

**Figure 9:**
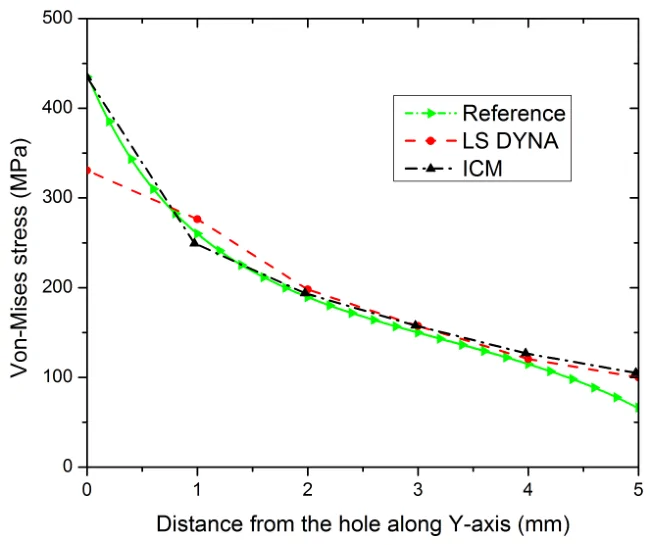
von Mises stress distribution along the Y-axis for G2 [39].

**Intermediate Refinement (G2):** With moderate mesh refinement (G2), standard FE solution errors remained unacceptably high, at −24.14% (Figure 8). In contrast, the ICM solution converged rapidly to the benchmark target, decreasing the localized stress error to +0.11%. Comparative stress profile curves along the domain centerline demonstrate excellent alignment between the ICM and the fine-mesh reference model (Figure 9).

**Figure 10:**
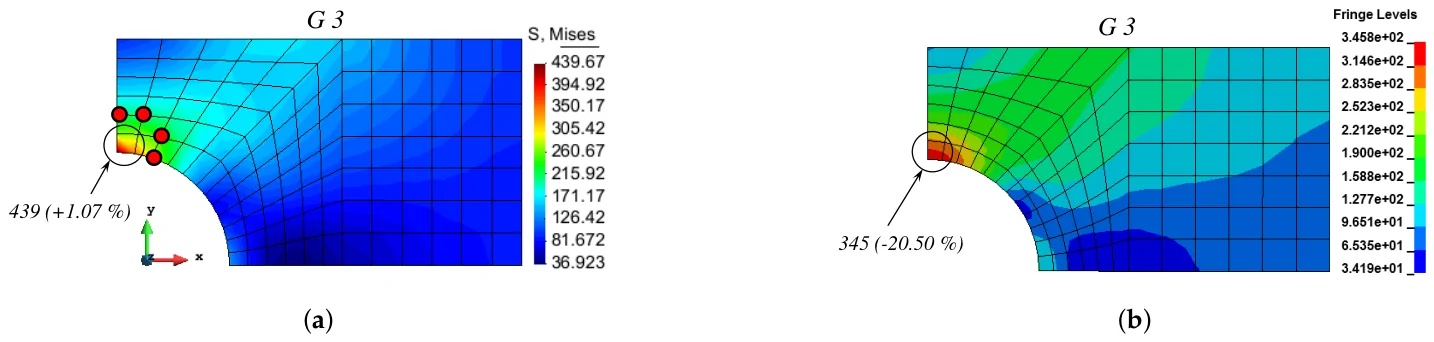
von Mises stress distribution along the Y-axis for G3 [39]. (**a**) ICM solution. (**b**) LS DYNA solution.

**Figure 11:**
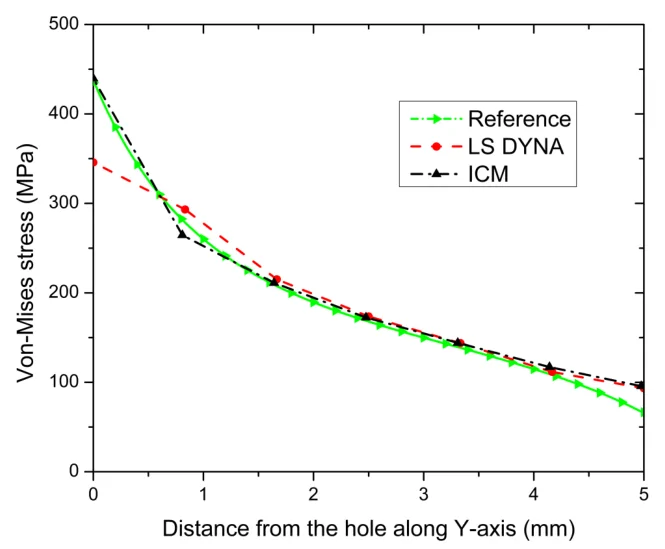
von Mises stress distribution along the Y-axis for G3 [39].

**Fine Discretization (G3):** For the G3 mesh layout, standard low-order quadrilateral elements continued to exhibit significant displacement locking and stress underestimation, with a −20.50% error (Figure 10). The proposed ICM formulation maintained a high level of accuracy, with an error of just +1.07%. As illustrated in the stress distribution plots (Figure 11), the ICM consistently captures the steep stress gradients near the geometric discontinuity without requiring additional global degrees of freedom or sub-element remeshing.

The von Mises stress comparison is shown in Table 1.

#### 3.1.1. Influence of Selection of the Neighboring Nodes

**Figure 12:**
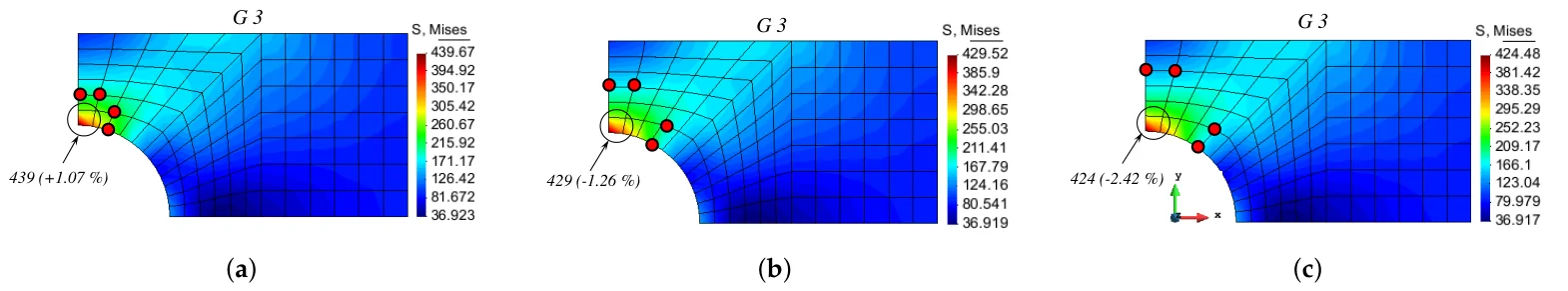
von Mises stress distribution: influence of neighboring nodes [39]. (**a**) 1st nearest neighboring nodes. (**b**) 2nd nearest neighboring nodes. (**c**) 3rd nearest neighboring nodes.

**Figure 13:**
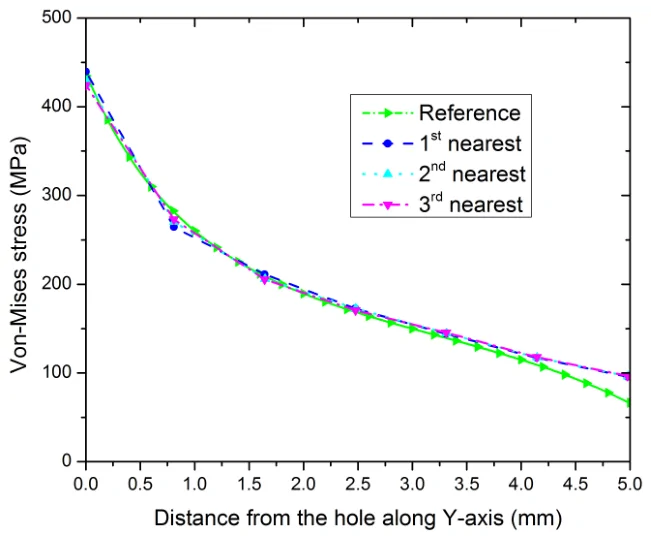
von Mises stress distribution along the Y-axis in G3 [39].

To evaluate the influence of surrounding node selection on the numerical performance of the ICM, a parametric study was conducted using the G3 enrichment scheme. Three distinct spatial configurations of neighboring nodes located in the immediate vicinity of the target element were selected for comparative analysis. The resulting von Mises stress distributions for each configuration are detailed in Figure 12. The von Mises stress profiles extracted along the Y-axis (Figure 13) demonstrate a clear dependency of the solution accuracy on the spatial arrangement of the enrichment nodes. Specifically, the numerical results indicate that optimal stress fields are obtained when selecting the first-nearest neighboring nodes relative to the target element. Incorporating more distant nodes introduces spatial bias, whereas prioritizing the closest local topology ensures superior accuracy in capturing high stress gradients.

#### 3.1.2. Influence of Selection of Enrichment Terms

In this section, the influence of the enrichment polynomial basis is discussed. In the above section, four additional polynomial terms were selected for enrichment, designated as Poly8. In Poly8, four additional terms were added to the four classical terms of the standard Q4 shell element. These additional terms are

(22) $${\tilde{N}}_{i}=N_{i}\;{\left\{\xi ,\;\eta ,\;\xi^{2}\eta ,\;\xi \eta^{2}\right\}}^{T}$$

These enrichment terms are extended to five in Poly9 as

(23) $${\tilde{N}}_{i}=N_{i}\;{\left\{\xi ,\;\eta ,\;\xi^{2}\eta ,\;\xi \eta^{2},\;\xi^{2}\eta^{2}\right\}}^{T}$$

In this case, five extra nodes need to select for enrichment. The third case is Poly10, where six additional enrichment terms are added, which are

(24) $${\tilde{N}}_{i}=N_{i}\;{\left\{\xi ,\;\eta ,\;\xi^{2}\eta ,\;\xi \eta^{2},\;\xi^{2}\eta^{2},\;\xi^{n}\eta^{n}\right\}}^{T}$$

where the power of *n* can be adjusted by the user to different values from n=3,4,…. In Poly10, six additional nodes need to be selected to enrich one element.

**Figure 14:**
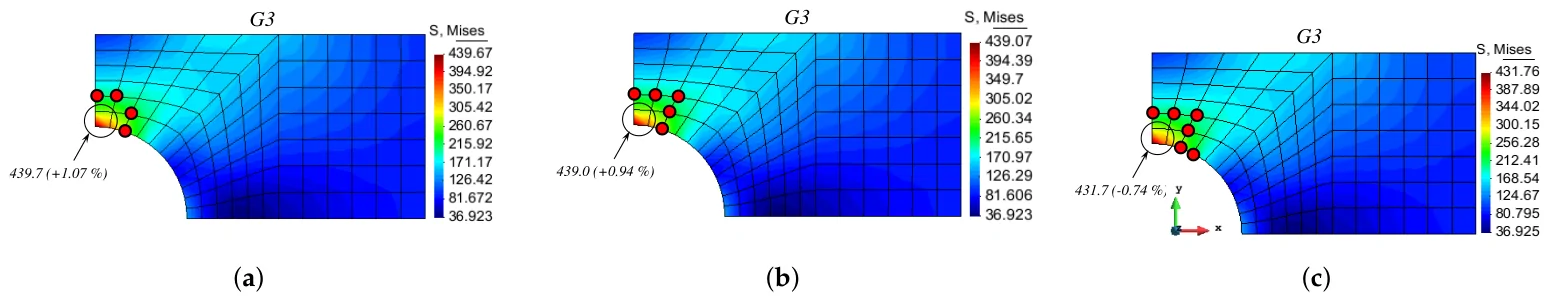
von Mises stress distribution: influence of polynomial enrichment bases [39]. (**a**) Poly8. (**b**) Poly9. (**c**) Poly10.

The plate-with-a-hole numerical application is selected to study all three enrichment cases of the ICM. A von Mises stress comparison between all the three cases is shown in Figure 14, where the maximum von Mises stress value doe not vary too much and the error percentage is up to 1% in all the cases. The highest value of von Mises stress is obtained by selecting four surrounding nodes in Poly8, whereas a slightly reduced von Mises value is obtained in Poly10, where six surrounding nodes are used for enrichment. Selecting surrounding nodes far from the enriched element results in a reduced von Mises stress value in Poly10.

The influence of the enrichment polynomial basis on the numerical response of the ICM formulation is investigated using the plate-with-a-hole benchmark. In the Poly8 formulation, four additional polynomial terms are introduced into the standard Q4 shell approximation, where additional nodes can be selected by the user according to the desired enrichment order. The number of surrounding nodes is selected consistently with the number of additional enrichment terms. Figure 14 compares the von Mises stress distributions obtained using Poly8, Poly9, and Poly10. The maximum von Mises stress shows only a small variation among the three enrichment bases, with the difference remaining approximately within 1% for the investigated case. The results indicate that increasing the polynomial order does not produce a significant deterioration of the numerical response for the considered enrichment configurations. The influence of the surrounding-node selection was also investigated. As shown in Figure 12 and Figure 13, the first, second, and third nearest neighboring-node configurations produce similar stress distributions, although a small variation in the maximum von Mises stress is observed. These results indicate that the enrichment response is relatively insensitive to the investigated variations in the surrounding-node configuration. However, the results also demonstrate that the location and number of surrounding nodes can have a small influence on the recovered stress field. It should be noted that higher-order polynomial enrichment can generally lead to linear dependence or ill conditioning if redundant enrichment functions or inappropriate surrounding-node configurations are introduced. In the present ICM implementation, the enrichment is applied locally through the selected surrounding-node clusters, and the number of surrounding nodes is selected consistently with the enrichment basis. The Poly8, Poly9, and Poly10 configurations considered here produced stable numerical solutions, and no singular system was encountered in the investigated benchmark cases.

### 3.2. Clamped Plate Bending

**Figure 15:**
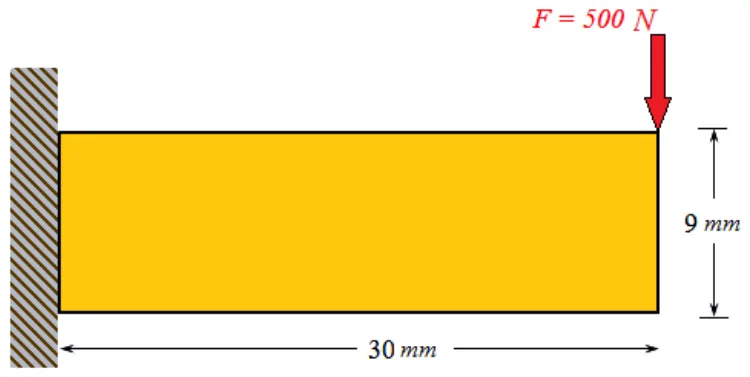
Clamped beam under application of transverse load [39].

The second solved application is a clamped plate under bending, as shown in Figure 15. A plane stress condition is assumed with material data of $E=72\times 10^{9}$ Pa, ν=0 and F=500 N with a thickness unity.

**Figure 16:**
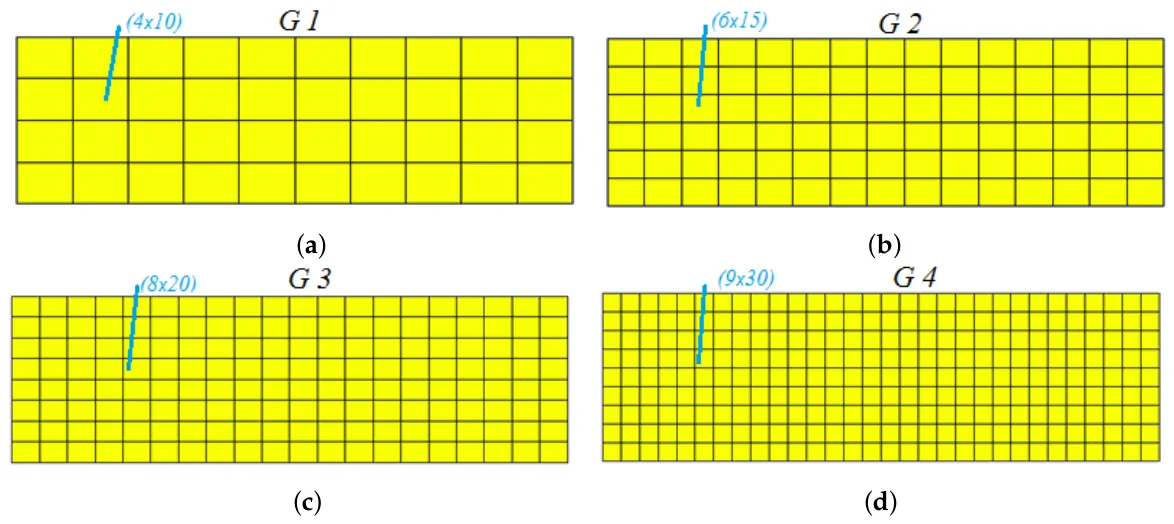
Clamped beam featuring four different mesh schemes [39]. (**a**) Total number of elements is 40. (**b**) Total number of elements is 90. (**c**) Total number of elements is 160. (**d**) Total number of elements is 270.

**Figure 17:**
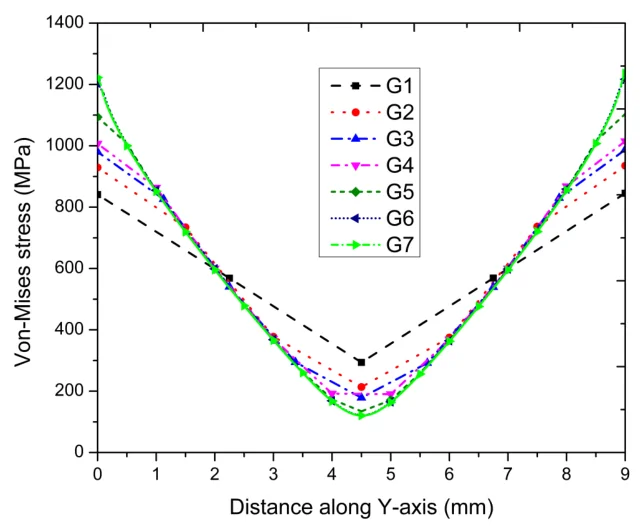
von Mises stress distribution of clamped beam along the Y-axis [39].

The beam is meshed with seven different mesh schemes via gradual refinement to find the converged solution. Four amongst seven meshes. along with their total numbers of elements, are presented in Figure 16. There are 1080, 27,000 and 108,000 elements used to model G5, G6 and G7 respectively. von Mises stress plots corresponding to all these seven mesh schemes are shown in Figure 17. the von Mises stress obtained by G7 with 108,000 total elements is taken as the reference solution.

A comparative numerical study was performed to assess the predictive accuracy of the ICM against standard FE formulations in LS-DYNA, using a high-fidelity reference solution for benchmark comparison. The evaluation focused on three distinct mesh configurations denoted as G1, G2, and G3 from the Figure 16, with local enrichment targeted at critical stress concentration zones near the clamped boundary.

**Figure 18:**
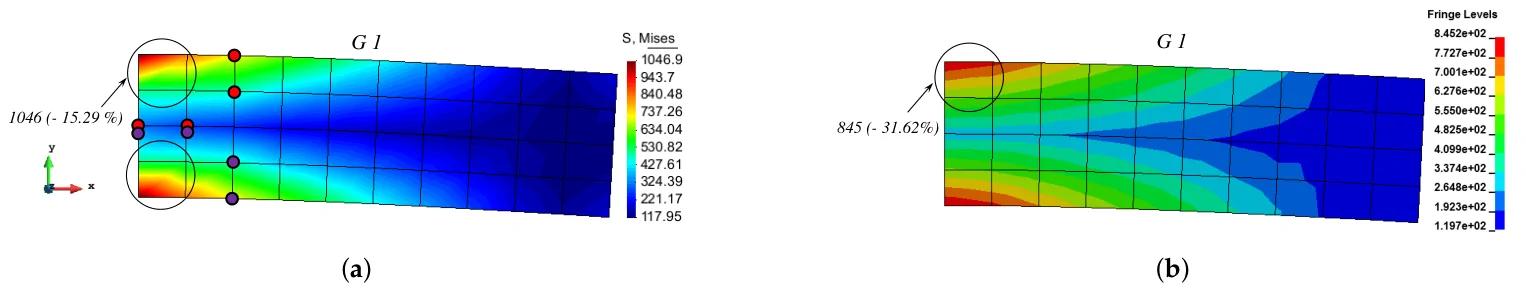
von Mises stress contour maps of clamped beam in G1 [39]. (**a**) ICM solution. (**b**) LS DYNA solution.

**Figure 19:**
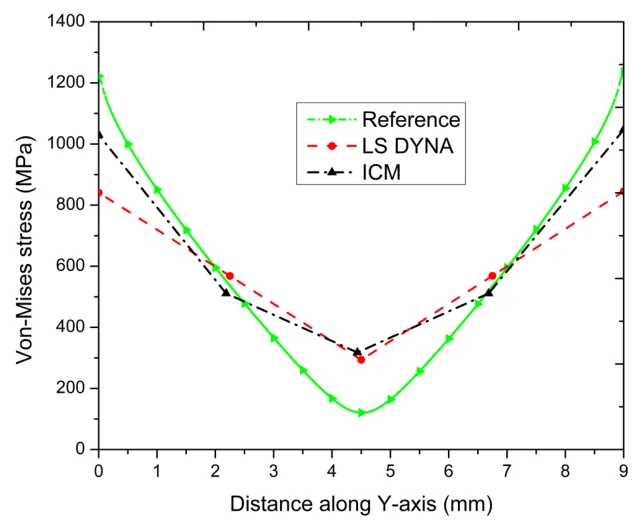
von Mises stress distribution of clamped beam along the Y-axis in G1 [39].

**Coarse Mesh Performance (G1):** Local cover enrichment was applied to two critical corner elements adjacent to the clamp, where maximum stress concentration occurs (indicated by black circles in Figure 18a). Each target element was enriched using four neighboring nodes. While the conventional LS-DYNA model severely underestimated the stress field, with a relative error of −31.62%, the ICM formulation substantially improved the solution, reducing the peak von Mises stress error to −15.29% (Figure 18 and Figure 19, respectively).

**Figure 20:**
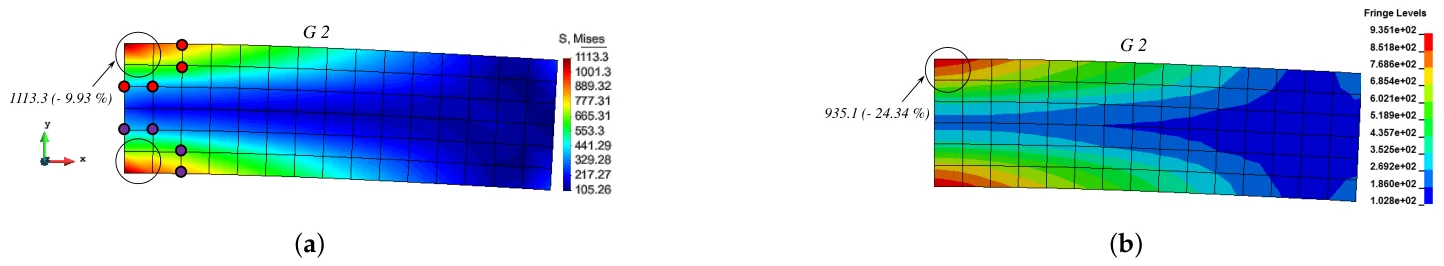
von Mises stress contour maps of clamped beam in G2 [39]. (**a**) ICM solution. (**b**) LS DYNA solution.

**Figure 21:**
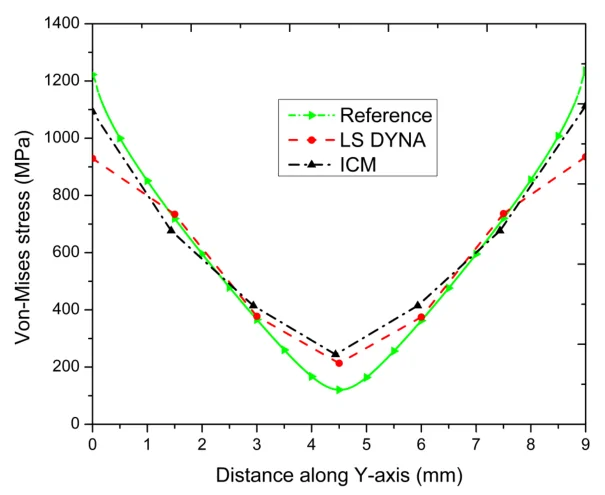
von Mises stress distribution of clamped beam along the Y-axis in G2 [39].

**Intermediate Refinement (G2):** Enriched cover functions in the G2 grid topology effectively captured the elevated stress gradients. The relative von Mises stress error dropped from −24.34% in standard LS-DYNA to −9.93% with the ICM, demonstrating improved spatial convergence along the Y-axis stress profile (Figure 20 and Figure 21).

**Figure 22:**
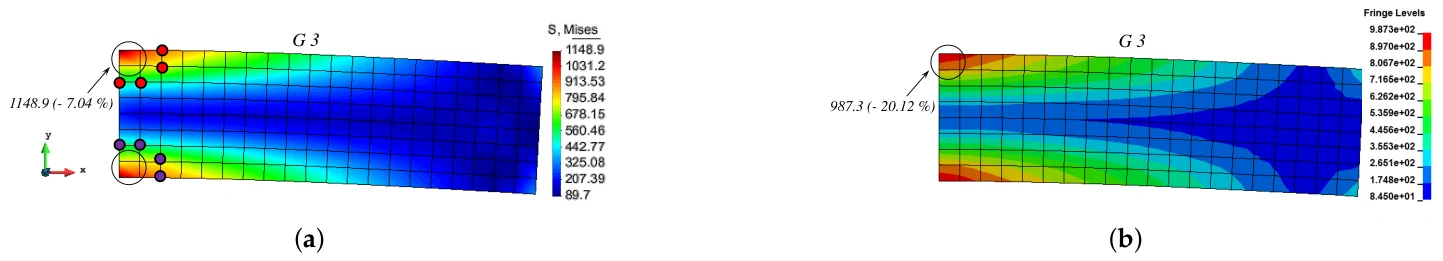
von Mises stress contour maps of clamped beam in G3 [39]. (**a**) ICM solution. (**b**) LS DYNA solution.

**Figure 23:**
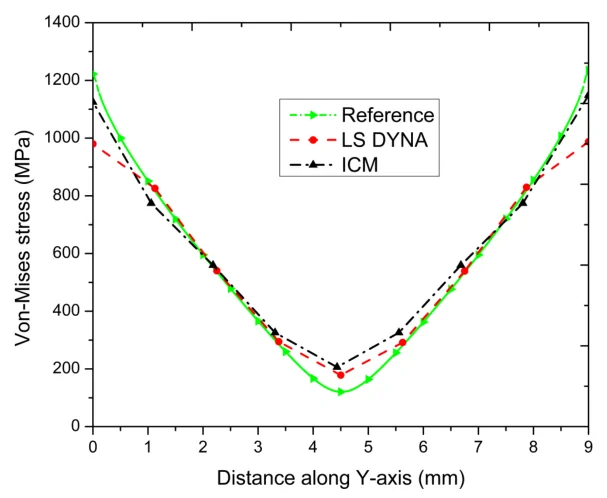
von Mises stress distribution of clamped beam along the Y-axis in G3 [39].

**Fine Discretization (G3):** The G3 discretization yielded the most refined numerical response. The relative error in peak von Mises stress was reduced from −20.12% (LS-DYNA) to −7.04% using the ICM, showing strong alignment with the reference profile (Figure 22 and Figure 23).

The von Mises stress comparison of the clamped plate-bending example is shown in Table 2.

#### 3.2.1. Influence of Selection of the Neighboring Nodes

**Figure 24:**
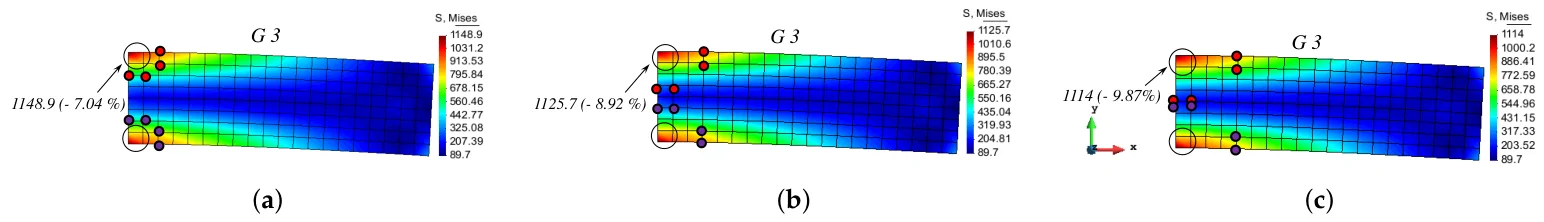
von Mises stress distribution: influence of surrounding nodes [39]. (**a**) 1st nearest neighboring nodes. (**b**) 2nd nearest neighboring nodes. (**c**) 3rd nearest neighboring nodes.

**Figure 25:**
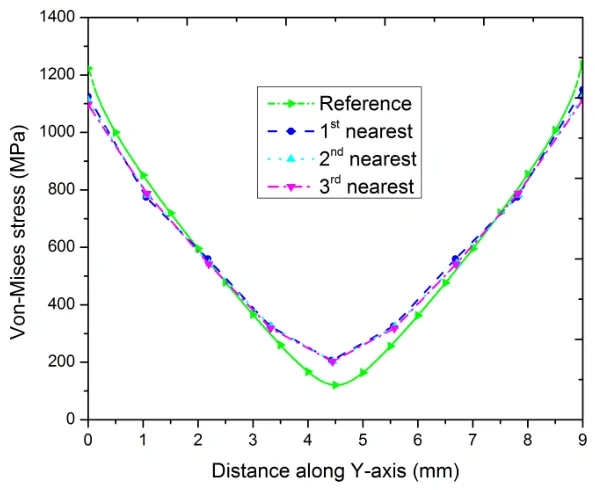
von Mises stress distribution along the Y-axis in G3 [39].

The influence of surrounding node selection on the predictive accuracy of the ICM was systematically evaluated using mesh configuration G3, adhering to the methodology established in the plate-with-a-hole benchmark. Three distinct spatial configurations of neighboring nodes were analyzed to enrich the target elements (Figure 24). The resulting von Mises stress profiles along the Y-axis were compared against both standard LS-DYNA predictions and the high-fidelity reference solution (Figure 25). Numerical results demonstrate that local stress recovery is highly sensitive to the spatial arrangement of the enrichment stencil:

First-Nearest Neighbor Selection: Incorporating the immediate (first-nearest) surrounding nodes relative to the target element yielded the highest peak von Mises stress values, producing the lowest relative error compared to the reference solution.
Distant Neighbor Selection: Progressively selecting neighboring nodes farther from the enriched element led to a monotonic decline in computed stress magnitudes, increasing solution attenuation and degrading overall enrichment performance. These observations confirm that compact, locally bounded enrichment stencils—formed by immediate topological neighbors—maximize interpolation accuracy and effectively capture steep stress gradients without introducing artificial spatial distortion.

The influence of neighboring-node selection can be interpreted from the locality of the interpolation-cover enrichment. In the present formulation, the enrichment functions are constructed in such a way that the additional interpolation terms introduce higher-order spatial variation into the standard bilinear approximation. When first-nearest neighboring nodes are selected, the interpolation cover remains localized around the target element, and the additional nodal information primarily represents the displacement field in its immediate vicinity. This is advantageous for capturing local displacement and stress gradients without introducing an unnecessarily large interpolation region. In contrast, selecting more distant neighboring nodes enlarges the spatial extent of the interpolation cover and reduces the locality of the enrichment. The resulting higher-order approximation is then influenced by nodal information from a larger region, which may be less representative of the local field surrounding the target element. Furthermore, the increased spatial separation of the participating nodes can lead to less favorable scaling and conditioning of the enriched interpolation system. These effects provide a mathematical interpretation of the observed reduction in accuracy for distant-node selections. Therefore, the superior performance of first-nearest neighboring nodes is attributed primarily to the improved locality of the interpolation cover, while the deterioration associated with distant nodes may additionally be accompanied by conditioning effects.

The quantitative results demonstrate that, although the enriched formulation increases the number of degrees of freedom at the element level and produces a somewhat wider matrix stencil, it avoids the generation and assembly of a globally refined sub-element mesh. Consequently, the proposed ICM formulation provides a favorable balance between local enrichment and global discretization complexity. In particular, the computational cost associated with the additional enrichment degrees of freedom remains lower than that associated with the equivalent globally h-refined FE model for the considered benchmark.

#### 3.2.2. Influence of Selection of Enrichment Terms

**Figure 26:**
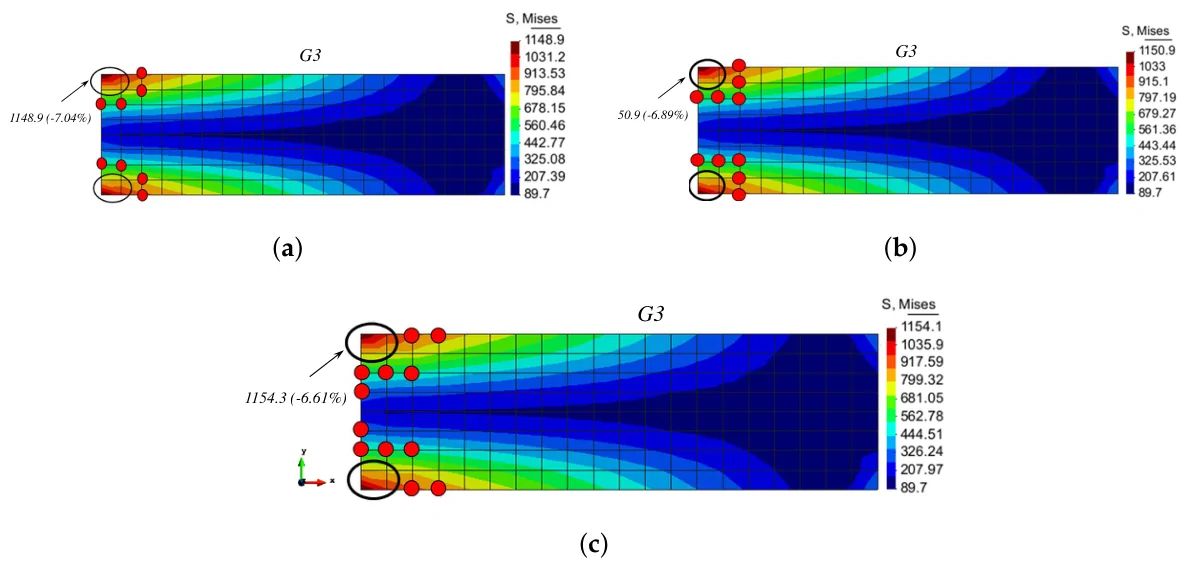
Comparison of stress distributions across Poly8, Poly9, and Poly10 clamped plates [39]. (**a**) Poly8. (**b**) Poly9. (**c**) Poly10.

The stress distributions and maximum von Mises stress values for the G3 refinement level across three polynomial orders (Poly 8, Poly 9, and Poly 10) are presented in Figure 26. The contours clearly illustrate the high-stress concentrations concentrated around the boundary constraints on the left-hand edge of the clamped plate. As shown in the comparative contours, increasing the polynomial order leads to a monotonic convergence toward the reference stress field. The Poly8 configuration predicts a peak von Mises stress of 1148.9MPa, corresponding to a relative error of −7.04%. The Poly9 configuration yields a peak stress of 1150.9MPa, reducing the relative error magnitude to −6.89%. The Poly10 configuration achieves the highest peak stress of 1154.3MPa, with the lowest error of −6.61%. The localized stress concentrations at the top and bottom re-entrant corners of the support boundary are captured progressively better as the polynomial degree increases from Poly8 to Poly10. The overall global stress field remains consistent across all three orders (ranging from minimum values around 89.7MPa at the right free edge to over 1140MPa near the boundary constraints), confirming mesh stability under higher-order shape functions. The steady reduction in relative error percentage from −7.04% to −6.61% demonstrates the effectiveness of *p*-refinement in capturing sharp boundary-layer stress gradients without requiring excessive local *h*-refinement.

The influence of selecting neighboring nodes in the plate-with-hole and clamped-beam examples is summarized in Table 3. The results indicate that the optimal polynomial enrichment order depends on the characteristics of the local solution field rather than increasing monotonically with polynomial degree. For the plate with a hole, the dominant feature is the localized stress concentration around the hole boundary. In this case, Poly8 provides a favorable balance between the ability to represent the local field variation and the additional complexity introduced by higher-order enrichment. For the clamped bending beam, the solution is governed primarily by bending deformation and the associated spatial variation of curvature and stress, for which the higher-order Poly10 enrichment provides improved accuracy. These results suggest that the polynomial order should be selected according to the complexity and spatial extent of the displacement and stress gradients generated by the geometry and loading conditions. As a practical guideline, a relatively lower-order enrichment can be considered for problems in which the dominant field variation is localized and can be adequately represented by the lower-order basis, whereas a higher-order enrichment may be advantageous when pronounced or more distributed field gradients are present. Therefore, Poly8, Poly9, and Poly10 should be regarded as alternative enrichment levels whose suitability depends on the characteristics of the specific engineering problem rather than as universally ranked schemes.

## 4. Conclusions

This study presented the Interpolation Covers Method (ICM) to enrich standard four-node shell elements, significantly enhancing solution accuracy without necessitating mesh refinement. By combining newly developed interpolation cover functions over element patches with standard finite element shape functions, the method effectively captures complex stress fields. Numerical evaluations against LS-DYNA and reference solutions demonstrate that the ICM achieves high stress accuracy, even on coarse meshes. Sensitivity analyses regarding node selection revealed that incorporating nodes farther from the enriched element degrades accuracy, emphasizing the importance of localized patch selection. Among the tested schemes, Poly8, Poly9, and Poly10, the suitability depends on the characteristics of the specific engineering problem rather than universally ranked schemes. The numerical investigations presented in this study are restricted to linear–elastic shell problems with small deformations.

## Figures and Tables

**Table 1:** von Mises stress comparison: plate with a hole [39].

Mesh Scheme	Element Count Q4	LS-DYNA (Standard FE) Error vs. Ref.	Proposed ICM Error vs. Ref.	Error Reduction
G1	Coarsest	−28.74%	−1.15% (Poly8)	27.59%
G2	Intermediate	−24.14%	+0.11% (Poly8)	24.03%
G3	Fine	−20.50%	+1.07% (Poly8)	19.43%

**Table 2:** von Mises stress comparison: clamped plate bending [39].

Mesh Scheme	Element Count Q4	LS-DYNA (Standard FE) Error vs. Ref.	Proposed ICM Error vs. Ref.	Error Reduction
G1	Coarsest	−31.62%	−15.29% (Poly8)	16.33%
G2	Intermediate	−24.34%	−9.93% (Poly8)	14.41%
G3	Fine	−20.12%	−7.04% (Poly8)	13.08%

**Table 3:** von Mises stress comparison: ICM vs. LS DYNA [39].

	Plate with a Hole	Error %	Approx. CPU Time	Clamped Beam	Error %	Approx. CPU Time
Reference	435		35 min	1236		20 min
LS DYNA	345.8	−20.5%	15 s	987.3	−20.12%	8 s
Poly8	439.67	+1.07%	30 s	1148.9	−7.04%	20 s
Poly9	439.07	+0.94%	35 s	1150.9	−6.89%	25 s
Poly10	431.76	−0.74%	45 s	1154.3	−6.61%	35 s

## Data Availability

The original contributions presented in this study are included in the article. Further inquiries can be directed to the corresponding authors.
